# Long-Term Use of Immunosuppressive Agents Increased the Risk of Fractures in Patients with Autoimmune Diseases: An 18-Year Population-Based Cohort Study

**DOI:** 10.3390/biomedicines11102764

**Published:** 2023-10-12

**Authors:** Feng-Chen Kao, Yao-Chun Hsu, Yuan-Kun Tu, Tzu-Shan Chen, Hsi-Hao Wang, Jeff (Chien-Fu) Lin

**Affiliations:** 1 Department of Orthopedics, E-Da Hospital, Kaohsiung City 824, Taiwan; kaofengchen@gmail.com (F.-C.K.); ed100130@edah.org.tw (Y.-K.T.); 2 School of Medicine, College of Medicine, I-Shou University, Kaohsiung City 824, Taiwan; holdenhsu@gmail.com (Y.-C.H.); hhgnaw@gmail.com (H.-H.W.); 3 Department of Orthopedics, E-Da Dachang Hospital, Kaohsiung City 807, Taiwan; 4 Division of Gastroenterology, E-Da Hospital, Kaohsiung City 824, Taiwan; 5 Department of Medical Research, E-Da Hospital, Kaohsiung City 824, Taiwan; ed107454@edah.orh.tw; 6 Department of Medical Imaging and Radiological Sciences, College of Medicine, I-Shou University, Kaohsiung City 824, Taiwan; 7 Division of Nephrology, Department of Internal Medicine, E-DA Hospital, Kaohsiung City 824, Taiwan; 8 Department of Medical Quality, E-DA Hospital, Kaohsiung City 824, Taiwan; 9 Department of Statistics, National Taipei University, Taipei City 23741, Taiwan; 10 Department of Orthopedic Surgery, Wan Fang Hospital, Taipei City 116, Taiwan

**Keywords:** immunosuppressive agents, autoimmune diseases, fracture, risk, cohort study

## Abstract

The risk of fractures is higher in patients with autoimmune diseases, but it is not clear whether the use of immunosuppressive agents can further increase this risk. To investigate this issue, a retrospective study was conducted using data from Taiwan’s National Health Insurance Research Database. Patients diagnosed with autoimmune diseases between 2000 and 2014, including psoriatic arthritis, rheumatoid arthritis, ankylosing spondylitis, and systemic lupus erythematosus, were included in the study. A control group of patients without autoimmune diseases was selected from the same database during the same period. Patients with autoimmune diseases were divided into two sub-cohorts based on their use of immunosuppressive agents. This study found the risk of fractures was 1.14 times higher in patients with autoimmune diseases than in those without. Moreover, we found that patients in the immunosuppressant sub-cohort had a higher risk of fractures compared to those in the non-immunosuppressant sub-cohort. The adjusted sub-distribution hazard ratio for shoulder fractures was 1.27 (95% CI = 1.01–1.58), for spine fractures was 1.43 (95% CI = 1.26–1.62), for wrist fractures was 0.95 (95% CI = 0.75–1.22), and for hip fractures was 1.67 (95% CI = 1.38–2.03). In conclusion, the long-term use of immunosuppressive agents in patients with autoimmune diseases may increase the risk of fractures.

## 1. Introduction

Autoimmune diseases are a group of disorders in which the immune system mistakenly attacks and damages healthy body tissues [1]. These diseases affect millions of people worldwide and can lead to significant disability and reduced quality of life [1]. Immunosuppressive agents are commonly used to manage the symptoms of autoimmune diseases by suppressing the immune system and reducing inflammation. However, there is growing concern that the use of these medications may increase the risk of bone fractures, a serious and potentially debilitating complication [2,3,4].

Several studies have investigated the association between immunosuppressive agents and fracture risk in patients with autoimmune diseases. For example, van Staa et al. conducted a retrospective cohort study and found that the long-term use of oral corticosteroids was associated with an increased risk of fractures in patients with rheumatoid arthritis [5]. In addition, Grijalva et al. found that the use of biologic DMARDs was associated with a decreased risk of hip fractures in patients with rheumatoid arthritis [6]. 

The mechanisms underlying the association between immunosuppressive agents and fracture risk in autoimmune disease patients are complex and not fully understood. However, it is thought that these medications may affect bone metabolism and increase the risk of osteoporosis, a condition characterized by low bone density and increased fracture risk [7]. Additionally, factors such as the type and dose of medication, duration of use, and underlying disease activity may play a role in fracture risk [2,3].

Given the potential for increased fracture risk associated with the use of immunosuppressive agents, it is important for clinicians to be aware of this potential complication and take appropriate measures to mitigate the risk. These measures may include the close monitoring of patients, lifestyle modifications to promote bone health, and the use of bone-protective medications, such as bisphosphonates or denosumab [8,9,10]. Furthermore, research is needed to better understand the mechanisms underlying the association between immunosuppressive agents and fracture risk in autoimmune disease patients, as well as to identify strategies to minimize this risk.

While immunosuppressive agents are important treatments for autoimmune diseases, they may increase the risk of bone fractures in these patients. Close monitoring and appropriate interventions can help mitigate this risk, but further research is needed to fully understand the mechanisms underlying this association and to identify effective strategies to minimize fracture risk in autoimmune disease patients.

The Taiwan National Health Insurance Research Database provides detailed information on the medication and medical history of a large number of patients, and thus, it is a valuable resource for investigation of the treatments and outcomes of many different diseases. Therefore, the aim of this study was to investigate whether the long-term use of immunosuppressive agents is related to the risk of fractures among patients with autoimmune diseases using a nationwide database. 

## 2. Materials and Methods

### 2.1. Data Source

This study was conducted using Taiwan’s National Health Insurance Research Database (NHIRD) [11], which was derived from the National Health Insurance (NHI) claims data with approximately 99.9% of Taiwan’s population (23 million). The enrollees in the database were de-identified by the Taiwan government, and the data were provided to scientists for research purposes. Study subjects were selected according to the diagnostic codes of the International Classification of Disease, Ninth Revision, Clinical Modification (ICD-9-CM), and International Classification of Diseases Tenth Revision (ICD-10) (Table A1 and Table A2). 

### 2.2. Identification of Autoimmune Disease

We selected patients who were newly diagnosed with psoriatic arthritis, rheumatoid arthritis, ankylosing spondylitis, or systemic lupus erythematosus between 1 January 2000 and 31 December 2014 as our study subjects. Subjects with at least one inpatient admission with a diagnosis of one of the selected diseases or at least three outpatient visits within a six-month period, each with a diagnosis of one of the selected diseases, were included in this study and defined as the “autoimmune disease cohort”, and the index day was defined as the diagnosis day. Subjects for the non-autoimmune disease cohort were selected from the NHIRD database during the same study period who were neither diagnosed with the selected diseases nor had any use of immunosuppressive agents. The autoimmune disease cohort and the non-autoimmune disease cohort were matched at a ratio of 1:4 according to age, gender, index date, and propensity scores of comorbidities.

### 2.3. Immunosuppressant Medication

Prescribed use of immunosuppressive agents in the follow-up period was also considered. The immunosuppressant medications used in this study included the conventional DMARDs cyclosporin, leflunomide, mycophenolate mofetil, methotrexate, and mycophenolic acid; the anti-TNF therapeutics adalimumab, certolizumab pegol, etanercept, infliximab, and golimumab; the mTOR inhibitors everolimus, sirolimus, and temsirolimus; the calcineurin inhibitor tacrolimus; and other biologics including abatacept (CTLA-4 analog), ixekizumab (anti-IL-17A), natalizumab (anti α-4 integrin), secukinumab (anti-IL-17), tocilizumab (anti-interleukin 6 [IL-6)], ustekinumab (anti-IL-12/IL-23), and vedolizumab (anti-α4β7 integrin). We collected data on the number of days each drug was prescribed within one year of diagnosis in Table A3.

Prescription records contained dates of order, dosage, the route of every prescription, and the number of days prescribed for each dispensed drug. Immunosuppressant medications were measured during the 365-day window between the diagnosis day and 365 days after the diagnosis day, and the index day was defined as the day 365 days after the diagnosis day. Moreover, patients with over 30 days of immunosuppressant medication were defined as the “immunosuppressant sub-cohort”, while those without any immunosuppressant medication during the 365-day window were defined as the “non-immunosuppressant sub-cohort”, and the index day was also defined as the day 365 days after the diagnosis day. Patients in the non-immunosuppressant sub-cohort who used immunosuppressants during the follow-up period were excluded. The immunosuppressant sub-cohort was matched (1:4) with the non-immunosuppressant sub-cohort according to age, gender, index date, and propensity scores of comorbidity. The index date for the immunosuppressant sub-cohort was assigned as one year after a newly selected disease diagnosis, while that for the non-immunosuppressant sub-cohort was assigned the date of the matched individuals. Steroids were also considered in the 365-day window, and subjects with over 90 days of medication were defined as regular users. Steroid use included prednisolone, methylprednisolone, budesonide, and cetylpyridinium/ephedrine.

### 2.4. Diagnosis of Fractures

The primary outcomes were patients diagnosed with shoulder fracture, spine fracture, wrist fracture, or hip fracture. Patients diagnosed with shoulder, spine, wrist, or hip fractures prior to the index date were excluded from this study. Fractures caused by accidents were excluded in this study.

### 2.5. Comorbidities

Comorbidities were classified as those existing prior to the index date. The baseline comorbid conditions were defined by ICD-9-CM and ICD-10 codes, including myocardial infarction, congestive heart failure, peripheral vascular disease, cerebrovascular disease, dementia, chronic lung disease, connective tissue disease, ulcer, chronic liver disease, diabetes, diabetes with end organ damage, hemiplegia, moderate or severe kidney disease, moderate or severe liver disease, tumor, leukemia, lymphoma, malignant tumor, metastasis, and acquired immune deficiency syndrome (AIDS).

### 2.6. Endpoint of the Follow-Up Period

The end of the follow-up period for the two analyses (i.e., autoimmune disease cohort versus non-autoimmune disease cohort and immunosuppressant sub-cohort versus non-immunosuppressant sub-cohort) was marked on the day of the shoulder, spine, wrist, or hip fracture diagnosis; terminated enrolment from NHI (31 December 2017); death; or the end of this study. Therefore, the maximum follow-up was 18 years (1 January 2000 to 31 December 2017). The minimum and median follow-up were 2 and 9.38 years, respectively. 

### 2.7. Statistical Analysis

Baseline characteristics and comorbidities for all cohorts were first analyzed. Typically, traditional survival analysis only considers one event at a time (e.g., death or fracture), possibly causing other events to be overlooked and resulting in overestimating risk. Thus, such results should not be directly interpreted and applied in clinical settings. To address this issue, our study considered death as a competing risk using the Fine and Gray sub-distribution proportional hazards model to calculate the sub-distribution hazard ratios (sHRs) and the Gray’s test. We compared the immunosuppressant and non-immunosuppressant sub-cohorts to the non-autoimmune disease cohort to evaluate the varying risks of developing five types of fracture after adjusting for age, gender, steroid use, and Charlson comorbidities. The adjusted sub-distribution hazard ratios (sHRs) with corresponding 95% confidence intervals (CIs) were calculated. The cumulative incidences of hip fracture between the immunosuppressant cohort and the non-immunosuppressant cohort were analyzed using competing risk analysis. All data management and analysis were conducted using SAS software (SAS System for Windows, Version 9.4; SAS Institute Inc., Cary, NC, USA). The Fine and Gray regression hazards model was employed using the PHREG package. *p*-Values less than 0.05 were considered statistically significant.

### 2.8. Ethics Statement

This study was approved by the Institutional Review Board of E-DA Hospital, Kaohsiung, Taiwan (Approval No. EMRP-108-061).

## 3. Results

### 3.1. Baseline Characteristics of Study Subjects

**Figure 1:**
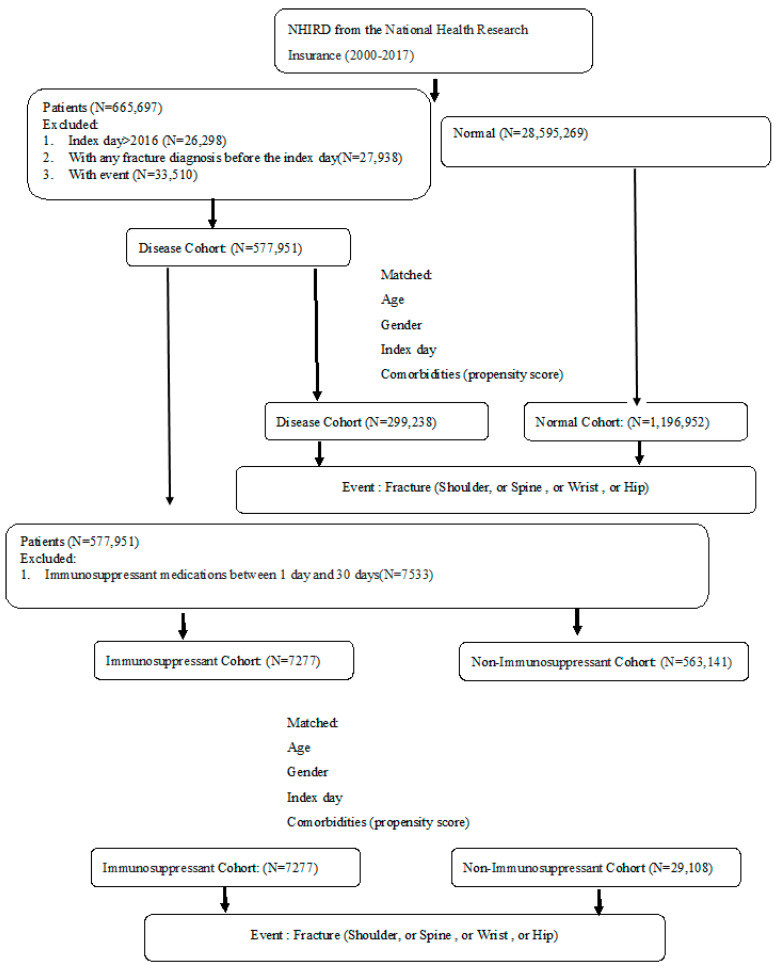
Study flow diagram.

From the NHIRD claims data, study subjects were matched in this study from 1 January 2000 to 31 December 2014 and were followed up until the end of 2017. A total of 299,238 patients in the autoimmune disease cohort and 1,196,952 subjects in the non-autoimmune disease cohort were matched by age, gender, index date, and comorbidities at a 1:4 ratio (Figure 1). Table 1 shows the mean age, gender, steroid medication, and Charlson comorbidities between the autoimmune disease and non-autoimmune disease cohorts after matching. A substantially higher proportion of patients with autoimmune disease had comorbid conditions including chronic lung disease, ulcers, and diabetes (Table 1). Among the patients with autoimmune disease, there were a total of 7277 patients with regular immunosuppressant usage in the immunosuppressant sub-cohort and 29,108 patients without any immunosuppressant medication in the non-immunosuppressant sub-cohort. These sub-cohorts were matched by age, gender, index date, and comorbidities at a 1:4 ratio. After matching, the baseline distributions were similar among the two sub-cohorts except for some comorbidities, such as congestive heart failure, dementia, ulcer, chronic liver disease, diabetes, and leukemia or lymphoma tumor (Table 2). 

### 3.2. The Risk of Fractures

**Figure 2:**
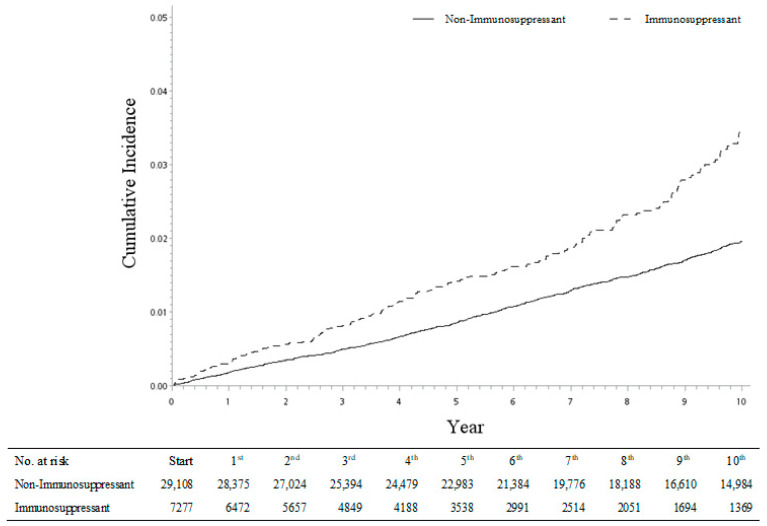
Cumulative incidences of hip fracture between immunosuppressant and non-immunosuppressant cohorts.

Based on the Fine and Gray model, the overall cumulative incidence of fracture was 1.14-fold higher in the autoimmune disease cohort than in the non-autoimmune disease cohort, including the shoulder, spine, wrist, and hip fractures (Figure 2). All the significant results were adjusted for age, gender, steroid medication, and Charlson comorbidities. When the autoimmune disease cohort was compared to the non-autoimmune cohort using the stratified Fine and Gray model, the adjusted sHR was 1.13 (95% CI = 1.10–1.17) for the risk of developing shoulder fracture, 1.94 (95% CI = 1.90–1.97) for the risk of developing spine fracture, 1.16 (95% CI = 1.13–1.19) for the risk of developing wrist fracture, and 1.14 (95% CI = 1.11–1.17) for the risk of developing hip fracture (Table 3). When the immunosuppressant sub-cohort was compared to the non-immunosuppressant sub-cohort using the stratified Fine and Gray model, the adjusted sHR was 1.27 (95% CI = 1.01–1.58) for the risk of developing shoulder fracture, 1.43 (95% CI = 1.26–1.62) for the risk of developing spine fracture, 0.95 (95% CI = 0.75–1.22) for the risk of developing wrist fracture, and 1.67 (95% CI = 1.38–2.03) for the risk of developing hip fracture (Table 4). 

Furthermore, with the same approach, we also analyzed the incidence risk of four types of fractures (shoulder, spine, wrist, and hip fracture) among each of the four autoimmune diseases (psoriatic arthritis, rheumatoid arthritis, ankylosing spondylitis, and systemic lupus erythematosus). Although some sHRs were observed with a lack of statistical significance with wider 95% CI ranges because of the smaller sample sizes, the fracture incidence trend was consistent across the different disease sub-groups. Thus, our study results were robust and confirmed by the re-analyses (Table 5). It is worth noting that the risk of hip fracture was relatively high, especially in patients with systemic lupus erythematosus receiving regular immunosuppressant medications (sHR: 3.34, 95% CI = 2.17–5.15; Table 5). The cumulative incidence of hip fracture was significantly higher in the immunosuppressant sub-cohort compared with the non-immunosuppressant sub-cohort (Figure 2).

## 4. Discussion

The present study investigated the association between immunosuppressant use and fracture risk in patients with autoimmune diseases, including psoriatic arthritis, rheumatoid arthritis, ankylosing spondylitis, and systemic lupus erythematosus. Our findings revealed a higher risk of fracture in the immunosuppressant sub-cohort compared to the non-immunosuppressant sub-cohort in each of the four autoimmune diseases. These results contradict the current opinion regarding rheumatoid arthritis that considers immunosuppressant use as fracture preventative. However, our study has several strengths, including the use of a nationwide database containing a large sample size with a long-term follow-up period, the estimation of incidence risk of different fracture types, and individual matching by propensity score to minimize selection bias.

The results of this study strongly suggest that immunosuppressant use increases the risk of fracture in patients with autoimmune diseases. Within the autoimmune disease cohort, our analysis found the immunosuppressant sub-cohort had a higher risk of fracture than the non-immunosuppressant sub-cohort. There were similar trends in each of the four autoimmune diseases, although the smaller sample sizes limited the significance in some groups. The prevailing opinion on rheumatoid arthritis typically regards the use of immunosuppressants as a means to prevent fractures [12]. However, our research results contradict this notion. Nevertheless, there is some evidence that the use of conventional DMARDs, such as methotrexate, sulfasalazine, hydroxychloroquine, azathioprine, and leflunomide, do impact upon bone mineral density in rheumatoid arthritis, where the use of more targeted biologics, such as anti-TNF (etanercept, adalimumab, golimumab, certolizumab), anti-IL6 receptor (tocilizumab), CTLA4 analog (abatacept), anti-CD 20 (rituximab), and JAK inhibitor (tofacitinib), do not [13]. 

As bone mineral density is often presumed to be related to the risk of fragility fractures [14], this can suggest differences in fracture risk between therapeutic approaches. Two studies investigated the use of abatacept, a CTLA-4 analog used by some of the patients in our cohort analysis. One of the studies compared abatacept to other biologic DMARDs in patients with rheumatoid arthritis and found increased bone mineral density at the femoral neck but no significant difference at the lumbar spine [15]. The other study compared conventional DMARD, anti-TNF, and abatacept therapies in patients with rheumatoid arthritis [13]. Bone mineral density decreased in the conventional DMARD group at the femoral neck and spine at L1–4 and L1–4 in the anti-TNF group but was preserved at both locations in the abatacept group [13]. In systemic lupus erythematosus, there have been contradictory studies with the use of hydroxychloroquine, a conventional DMRD. Some studies in women found increased bone mineral density in either the lumber spine or hip [16,17], while mixed-sex populations found decreased bone mineral density [18,19] or no change [20]. Rethi Raghu Nadhanan et al. [21] suggested that emu oil supplementation can effectively combat inflammation, osteoporosis, and bone loss induced by 5-fluorouracil chemotherapy, enhancing patients’ overall quality of life. Meanwhile, Tristan J King et al. [22] highlighted the potential of genistein in reducing bone damage from methotrexate chemotherapy. In summary, these studies offer promising avenues to reduce bone loss during cancer treatment. Emu oil and genistein may hold the key to preserving bone health in chemotherapy patients, but further research is necessary to optimize their use. These findings inform future research and clinical practices for safeguarding the bone health of cancer patients.

Despite these bone mineral density results suggesting a relationship between DMRDs and the potential for fragility fractures, studies that directly investigated the risk of nonvertebral fractures in rheumatoid arthritis found no increase in risk with conventional DMARD use [20,23] and no decreased risk with the use of a variety of targeted or biologic DMARDs [23,24,25]. However, a meta-analysis of 100 randomized controlled trials suggested that, in patients with psoriatic arthritis, there was a decreased risk of major osteoporotic fracture, hip fracture, and osteoporotic nonvertebral fracture with biologic DMARDs [26]. That analysis did not identify any change in risk in patients with rheumatoid arthritis, axial spondylarthritis, systemic lupus erythematosus, or inflammatory bowel disease with biologic DMARD treatment [26]. These differences between bone mineral density and fracture risk might be explained in part by the limited ability of bone mineral density to directly predict the risk of fractures [4]. Nevertheless, they contradict the findings of our study that did show an increased risk of fractures across the autoimmune diseases investigated, with particular risk to the hip. This might be because these studies were conducted with smaller sample sizes or shorter follow-up periods compared to our study.

In Taiwan, physicians prescribe and adjust immunosuppressive agents based on the guidance issued by the Taiwan National Health Insurance Administration. We recommend that physicians evaluate risk factors of fracture before the administration of immunosuppressive agents and then perform osteoporosis assessment regularly after the administration of immunosuppressive agents for those patients with autoimmune diseases. We believe that our recommendation will assist physicians in ascertaining whether supplementary anti-osteoporotic treatments are necessary for those patients.

In this study, a wide range of immunosuppressive agents were used by the patients, including both conventional DMARDs and targeted biologics to receptors, cytokines, and other inflammatory molecules. Even since the introduction of biologic DMARDs in the early 1990s, most treatment strategies have prescribed conventional DMARDs, with biologic or targeted DMARDs considered only after treatment failure [27]. Previous studies have suggested there is no difference in the occurrence of nonvertebral osteoporotic fractures in patients with rheumatoid arthritis treated with the biologic or targeted DMARDs adalimumab, abatacept, certolizumab, etanercept, golimumab, infliximab, rituximab, tocilizumab, or tofacitinib [28] or with abatacept or tocilizumab compared to anti-TNF therapy [29]. Further study is needed to evaluate whether there is a difference in fracture risk between patients with long-term use of conventional or biologic/targeted DMARDs in this study population.

This study has several strengths. First, we used a nationwide database containing over 99.9% of the Taiwanese population, which allowed us to investigate a huge sample size with a long-term follow-up period. Second, there is limited research on the use of immunosuppressive drugs and fracture risk; this study assessed the risk of fractures among patients with one of four different autoimmune diseases (psoriatic arthritis, rheumatoid arthritis, ankylosing spondylitis, and systemic lupus erythematosus), who frequently use immunosuppressive drugs. Third, the incidence risks of four different fracture types (shoulder, spine, wrist, and hip fracture) were also estimated separately to understand which one presents the highest risk. Finally, the study subjects in the main cohort and sub-cohort were matched individually according to the propensity score to minimize the selection bias.

The current study has some limitations that should be considered when interpreting the results. First, the retrospective design of the study might have introduced some bias, although we attempted to minimize this by matching patients according to the propensity score. Second, our study did not assess the dose or duration of immunosuppressive use or the clinical stage of autoimmune diseases, which may influence fracture risk. Third, our study did not account for potential confounding factors, such as smoking status, alcohol consumption, and physical activity, which may affect fracture risk. Fourth, our study subjects were patients with selected autoimmune diseases, not all kinds of autoimmune diseases. Fifth, based on our data, the number of individuals exclusively receiving single-drug therapy is extremely limited. This significantly impairs the study’s quality. A comprehensive answer to this research question can be obtained through a well-designed clinical trial or a larger database than NHIRD. Finally, our study only included patients with autoimmune diseases, and the generalizability of our findings to other populations is unknown.

## 5. Conclusions

In conclusion, the results of this study suggest that immunosuppressive use may increase the risk of fractures in patients with autoimmune diseases, particularly at the hip. Clinicians should be aware of this potential risk and take steps to minimize the risk of falls in these patients. Further research is needed to elucidate the mechanisms underlying the association between immunosuppressant use and fracture risk in autoimmune diseases and to determine the optimal strategies for preventing fractures in these patients.

## Figures and Tables

**Table 1:** Baseline characteristics of the matched-study population (normal cohort and disease cohort).

	Normal N = 1,196,952	Disease N = 299,238	*p*-Value
Age (years)	48.32 ± 17.57	48.32 ± 17.57	0.9802
Gender			>0.9999
Female	581,488(48.58)	145,372(48.58)	
Male	615,464(51.42)	153,866(51.42)	
Medication			
Steroid	164,524(13.75)	79,733(26.65)	<0.0001
Comorbidities			
Myocardial infarct	23,982(2.00)	7133(2.38)	<0.0001
Congestive heart failure	36,852(3.08)	10,277(3.43)	<0.0001
Peripheral vascular disease	18,480(1.54)	5070(1.69)	<0.0001
Cerebrovascular disease	89,567(7.48)	26,252(8.77)	<0.0001
Dementia	10,446(0.87)	2913(0.97)	<0.0001
Chronic lung disease	287,834(24.05)	75,377(25.19)	<0.0001
Connective tissue disease	35,916(3.00)	8979(3.00)	>0.9999
Ulcer	419,536(35.05)	105,403(35.22)	0.0754
Chronic liver disease	206,112(17.22)	51,485(17.21)	0.8523
Diabetes	135,057(11.28)	37,785(12.63)	<0.0001
Diabetes with end organ damage	38,287(3.20)	10,914(3.65)	<0.0001
Hemiplegia	9054(0.76)	2267(0.76)	0.9473
Moderate or severe kidney disease	49,713(4.15)	13,664(4.57)	<0.0001
Tumor, leukemia, lymphoma	46,102(3.85)	13,504(4.51)	<0.0001
Moderate or severe liver disease	3479(0.29)	825(0.28)	0.1719
Malignant tumor, metastasis	6939(0.58)	1977(0.66)	<0.0001
AIDS	457(0.04)	91(0.03)	0.0470
Event			
Shoulder fracture	19,073(1.59)	5508(1.84)	<0.0001
Spine fracture	34,498(2.88)	16,989(5.68)	<0.0001
Wrist fracture	20,569(1.72)	6060(2.03)	<0.0001
Hip fracture	22,101(1.85)	6296(2.10)	<0.0001

**Table 2:** Baseline characteristics of the matched-study population (non-immunosuppressant cohort and immunosuppressant cohort).

	Non-Immunosuppressant N = 29,108	Immunosuppressant N = 7277	*p*-Value
Age (years)	46.2 ± 16.86	46.2 ± 16.86	0.999
Gender			>0.9999
Female	17,460(59.98)	4365(59.98)	
Male	11,648(40.02)	2912(40.02)	
Medication			
Steroid	11,051(37.97)	5341(73.4)	<0.0001
Comorbidities			
Myocardial infarct	520(1.79)	136(1.87)	0.6364
Congestive heart failure	858(2.95)	262(3.60)	0.0039
Peripheral vascular disease	472(1.62)	125(1.72)	0.5634
Cerebrovascular disease	1785(6.13)	497(6.83)	0.0282
Dementia	102(0.35)	42(0.58)	0.0059
Chronic lung disease	7516(25.82)	1857(25.52)	0.5979
Connective tissue disease	18,171(62.43)	4603(63.25)	0.1917
Ulcer	10,338(35.52)	2604(35.78)	0.6693
Chronic liver disease	5403(18.56)	1447(19.88)	0.0098
Diabetes	3283(11.28)	906(12.45)	0.0051
Diabetes with end organ damage	956(3.28)	255(3.50)	0.3497
Hemiplegia	163(0.56)	43(0.59)	0.7532
Moderate or severe kidney disease	2866(9.85)	722(9.92)	0.8466
Tumor, leukemia, lymphoma	1374(4.72)	368(5.06)	0.2289
Moderate or severe liver disease	152(0.52)	28(0.38)	0.1351
Malignant tumor, metastasis	184(0.63)	40(0.55)	0.4212
AIDS			
Event			
Shoulder fracture	532(1.83)	105(1.44)	0.0252
Spine fracture	1518(5.22)	331(4.55)	0.0206
Wrist fracture	597(2.05)	80(1.10)	<0.0001
Hip fracture	596(2.05)	150(2.06)	0.9410

**Table 3:** Sub-distribution hazard ratios of the risk factors associated with the occurrence of fractures based on Fine and Gray (sub-distribution hazard) model from competing risk analysis between autoimmune disease and non-autoimmune cohorts.

	Adjusted HRs			
	Shoulder	Spine	Wrist	Hip
Disease vs. normal	1.13(1.10–1.17)	1.94(1.90–1.97)	1.16(1.13–1.19)	1.14(1.11–1.17)
Age	1.02(1.02–1.02)	1.03(1.03–1.03)	1.03(1.03–1.03)	1.09(1.09–1.09)
Male vs. female	0.92(0.90–0.94)	0.78(0.76–0.79)	0.44(0.43–0.45)	0.76(0.74–0.78)
Steroid	1.10(1.07–1.14)	1.26(1.24–1.29)	1.11(1.08–1.15)	1.08(1.05–1.11)
Comorbidities				
Myocardial infarct	1.08(1.00–1.16)	1.13(1.08–1.19)	0.94(0.86–1.01)	0.98(0.93–1.04)
Congestive heart failure	1.07(1.00–1.14)	1.04(1.00–1.08)	0.94(0.88–1.00)	1.16(1.11–1.21)
Peripheral vascular disease	1.04(0.94–1.14)	1.23(1.16–1.30)	1.00(0.91–1.09)	1.14(1.07–1.22)
Cerebrovascular disease	1.11(1.06–1.16)	1.07(1.04–1.10)	1.01(0.97–1.05)	1.23(1.19–1.27)
Dementia	1.01(0.89–1.15)	0.58(0.53–0.64)	0.77(0.68–0.88)	1.21(1.13–1.30)
Chronic lung disease	1.10(1.07–1.14)	1.19(1.16–1.21)	1.14(1.11–1.17)	1.09(1.07–1.12)
Connective tissue disease	1.03(0.96–1.10)	1.20(1.15–1.25)	1.09(1.03–1.15)	1.06(1.01–1.12)
Ulcer	1.13(1.09–1.16)	1.48(1.45–1.51)	1.13(1.10–1.16)	1.08(1.05–1.10)
Chronic liver disease	1.09(1.06–1.13)	1.26(1.23–1.29)	1.12(1.09–1.16)	1.03(1.00–1.06)
Diabetes	1.26(1.21–1.31)	1.26(1.23–1.29)	1.09(1.05–1.13)	1.29(1.25–1.34)
Diabetes with end organ damage	1.25(1.17–1.33)	1.09(1.04–1.13)	1.08(1.01–1.15)	1.30(1.24–1.36)
Hemiplegia	1.06(0.93–1.22)	0.80(0.72–0.89)	0.70(0.59–0.82)	1.26(1.15–1.38)
Moderate or severe kidney disease	1.08(1.02–1.15)	1.12(1.08–1.16)	1.00(0.95–1.06)	1.17(1.13–1.22)
Tumor, leukemia, lymphoma	1.02(0.96–1.09)	1.04(1.00–1.08)	1.05(0.98–1.11)	1.04(0.99–1.09)
Moderate or severe liver disease	1.53(1.22–1.90)	0.90(0.76–1.08)	1.27(0.99–1.62)	2.28(1.94–2.69)
Malignant tumor, metastasis	0.95(0.77–1.16)	1.11(0.98–1.25)	1.14(0.96–1.36)	1.12(0.96–1.29)
AIDS	1.43(0.72–2.87)	0.75(0.36–1.56)	0.22(0.03–1.58)	1.85(0.83–4.12)

**Table 4:** Sub-distribution hazard ratios of the risk factors associated with the occurrence of fractures based on Fine and Gray (sub-distribution hazard) model from competing risk analysis between immunosuppressant and non-immunosuppressant cohorts.

	Adjusted HRs			
	Shoulder	Spine	Wrist	Hip
Immunosuppressant vs. non-immunosuppressant	1.27(1.01–1.58)	1.43(1.26–1.62)	0.95(0.75–1.22)	1.67(1.38–2.03)
Age	1.02(1.02–1.03)	1.04(1.04–1.04)	1.04(1.03–1.04)	1.07(1.06–1.07)
Male vs. female	1.11(0.94–1.31)	0.85(0.77–0.94)	0.57(0.47–0.68)	0.88(0.75–1.03)
Steroid	1.15(0.97–1.36)	1.26(1.14–1.39)	0.95(0.80–1.11)	1.36(1.16–1.59)
Comorbidities				
Myocardial infarct	1.33(0.84–2.11)	1.15(0.88–1.50)	0.81(0.46–1.42)	1.24(0.87–1.78)
Congestive heart failure	1.52(1.05–2.18)	1.13(0.91–1.40)	1.13(0.77–1.65)	1.50(1.15–1.96)
Peripheral vascular disease	1.25(0.73–2.14)	1.23(0.91–1.65)	0.82(0.44–1.54)	1.57(1.07–2.31)
Cerebrovascular disease	1.08(0.80–1.44)	0.94(0.80–1.11)	0.89(0.66–1.19)	1.03(0.82–1.30)
Dementia	0.97(0.31–3.06)	0.52(0.25–1.11)	1.95(0.92–4.16)	1.60(0.91–2.81)
Chronic lung disease	0.91(0.76–1.10)	1.10(0.99–1.22)	1.19(1.01–1.41)	1.08(0.92–1.27)
Connective tissue disease	1.17(0.97–1.41)	0.89(0.80–0.99)	0.98(0.82–1.18)	1.12(0.94–1.34)
Ulcer	1.30(1.10–1.54)	1.53(1.39–1.69)	1.05(0.90–1.24)	1.06(0.91–1.23)
Chronic liver disease	1.02(0.84–1.25)	1.19(1.07–1.33)	1.09(0.90–1.33)	0.92(0.76–1.11)
Diabetes	1.17(0.90–1.51)	1.28(1.12–1.47)	0.93(0.72–1.20)	1.25(1.02–1.54)
Diabetes with end organ damage	1.32(0.88–1.97)	0.97(0.76–1.22)	1.23(0.81–1.86)	1.28(0.93–1.76)
Hemiplegia	1.02(0.37–2.77)	1.07(0.60–1.91)	1.54(0.63–3.77)	1.81(0.95–3.44)
Moderate or severe kidney disease	1.07(0.83–1.38)	0.99(0.86–1.16)	0.89(0.68–1.16)	1.43(1.17–1.75)
Tumor, leukemia, lymphoma	0.89(0.60–1.32)	1.05(0.86–1.29)	0.85(0.58–1.25)	1.08(0.80–1.45)
Moderate or severe liver disease	1.06(0.34–3.32)	0.90(0.45–1.81)	1.69(0.63–4.56)	1.98(0.93–4.21)
Malignant tumor, metastasis	0.77(0.18–3.18)	1.52(0.89–2.60)	1.09(0.34–3.54)	1.82(0.87–3.81)
AIDS	NA	NA	NA	NA

**Table 5:** Sub-distribution hazard ratios of the risk factors associated with the occurrence of fractures based on Fine and Gray (sub-distribution hazard) model from competing risk analysis among four different types of autoimmune diseases.

	Adjusted HRs			
	Shoulder	Spine	Wrist	Hip
Disease vs. Normal				
Psoriatic arthritis	1.33(1.13–1.55)	1.44(1.29–1.62)	1.29(1.09–1.53)	1.38(1.17–1.62)
Rheumatoid arthritis	1.10(1.05–1.16)	1.70(1.64–1.75)	1.16(1.10–1.21)	1.19(1.13–1.24)
Ankylosing spondylitis	1.14(1.10–1.19)	2.16(2.11–2.21)	1.15(1.10–1.19)	1.10(1.06–1.14)
Systemic lupus erythematosus	0.80(0.48–1.31)	0.84(0.63–1.14)	1.20(0.83–1.75)	1.23(0.83–1.83)
Immunosuppressant vs. Non-immunosuppressant				
Psoriatic arthritis	0.83(0.33–2.09)	1.14(0.52–2.50)	0.78(0.30–2.07)	2.17(0.81–5.84)
Rheumatoid arthritis	1.45(1.11–1.88)	1.55(1.34–1.79)	0.85(0.63–1.16)	1.38(1.09–1.74)
Ankylosing spondylitis	1.13(0.61–2.08)	1.29(0.93–1.77)	0.63(0.28–1.38)	1.72(0.92–3.22)
Systemic lupus erythematosus	0.84(0.44–1.60)	1.71(1.16–2.52)	0.74(0.37–1.51)	3.34(2.17–5.15)

## Data Availability

https://dep.mohw.gov.tw/DOS/cp-5283-63826-113.html (accessed on 20 August 2023).

## Appendix A

**Table A1:** ICD-9-CM and ICD-10 diagnosis codes.

	ICD-9-CM	ICD-10
Shoulder fracture	810.xx (xx = 00~03; 10~13), 811.xx (xx = 00~03; 10~13; 09; 19), 812.aa (aa = 00~03; 10~13; 09; 19)	S42.00aA (a = 1,2,9) S42.0abA (ab = 11~26; 31~36) S42.00aB (a = 1,2,9) S42.0abB (ab = 11~26; 31~36) S42.10aA (a = 1,2,9) S42.1abA (ab = 21~26; 31~36; 41~46; 51~56) S42.11aA (a = 1~6) S42.19bA (b = 1;2;9) S42.10aB (a = 1,2,9) S42.1abB (ab = 21~26; 31~36; 41~46; 51~56) S42.11aB (a = 1~6) S42.19bB (b = 1;2;9) S42.20aA (a = 1,2,9) S42.2abA (ab = 11~16; 21~26; 31;32;39; 41;42;49; 51~56; 61~66; 71;72;79; 91~96) S49.0abA (a = 1;2;3;4;9; b = 1;2;9) S42.20aB (a = 1,2,9) S42.2abB (ab = 11~16; 21~26; 31;32;39; 41;42;49; 51~56; 61~66)
Elbow fracture	812.xx (xx = 40~44; 50~54; 49; 59) 813.xx (xx = 00~08; 10~18)	S42.40aA (a = 1,2,9) S42.4abA (ab = 11~16; 21~26; 31~36; 41~49; 51~56; 61~66; 71~76) S49.1abA (a = 0;1;2;3;4;9; b = 1;2;9) S42.48aA (a = 1;2;9) S42.49bA (b = 1~6) S42.40aB (a = 1,2,9) S42.4abB (ab = 11~16; 21~26; 31~36; 41~49; 51~56; 61~66; 71~76; 91~96) S52.90XA S52.0abA (ab = 21~26; 31~36; 41~46) S52.27aA (a = 1,2,9) S52.00aA (a = 1,2,9) S52.09aA (a = 1,2,9) S52.12aA (a = 1,~6) S52.13aA (a = 1,~6) S52.10aA (a = 1,2,9) S52.18aA (a = 1,2,9) S59.1abA (a = 0~4; 9; b = 1,2,9) S52.90XB S52.0abB (ab = 21~26; 31~36; 41~46) S52.27aB (a = 1,2,9) S52.00aB (a = 1,2,9) S52.09aB (a = 1,2,9) S52.1abB (ab = 21~26; 31~36) S52.10aB (a = 1,2,9) S52.18aB (a = 1,2,9) S52.90XC S52.0abC (ab = 21~26; 31~36; 41~46) S52.27aC (a = 1,2,9) S52.00aC (a = 1,2,9) S52.09aC (a = 1,2,9) S52.1abC (ab = 21~26; 31~36) S52.10aC (a = 1,2,9) S52.18aC (a = 1,2,9)
Wrist fracture	813.xx (xx = 40~44; 50~54) 814.xx (xx = 00~09; 10~19)	S52.90XA S52.53aA (a = 1,2,9) S52.54aA (a = 1,2,9) S52.11aA (a = 1,2) S52.51aA (a = 1~6) S52.5abA (a = 0; 2~7; 9; b = 1; 2; 9) S59.2abA (a = 0~4; 9; b = 1,2,9) S52.01aA (a = 1,2) S52.6abA (a = 0;2;9; b = 1,2,9) S52.61aA (a = 1~6) S59.0abA (a = 0;2;3;4;9; b = 1,2,9) S59.11aA (a = 1,2,9) S62.10aA (a = 1,2,9) S62.00aA (a = 1,2,9) S62.0abA (a = 1~3; b = 1~6) S62.1abA (a = 1~8; b = 1~6) S52.90XB S52.5abB (a = 0; 3~7; 9; b = 1,2,9) S52.51aB (a = 1~6) S52.6abB (a = 0; 9; b = 1;2;9) S52.6aB (a = 1~6) S62.10aB (a = 1,2,9) S62.00aB (a = 1,2,9) S62.0abB (a = 1~3; b = 1~6) S62.1abB (a = 1~8; b = 1~6) S52.90XC S52.5abC (a = 0; 3~7; 9; b = 1,2,9) S52.51aC (a = 1~6) S52.6abC (a = 0; 9; b = 1;2;9) S52.6aC (a = 1~6)
Hand fracture	815.xx (xx = 00~04; 10~14; 09; 19) 816.xx (xx = 00~03; 10~13) 817.0; 817.1	S52 S62.3a9A (a = 0~5;9) S62.20aA (a = 1,2,9) S62.21aA (a = 1,2,3) S62.2abA (a = 2~5; b = 1~6) S62.3abA (a = 0~6; 9; b = 0~9) S62.29aA (a = 1,2,9) S62.50aA (a = 1,2,9) S62.5abA (a = 1~2; b = 1~6) S62.6abA (a = 0~6; b = 0~9) S62.9aXA (a = 0;1,2) S62.309B S62.20aB (a = 1,2,9) S62.21aB (a = 1,2,3) S62.2abB (a = 2~5; b = 1~6) S62.3abB (a = 0~6; 9; b = 0~9) S62.29aB (a = 1,2,9) S62.50aB (a = 1,2,9) S62.5abB (a = 1~2; b = 1~6) S62.6abB (a = 0~6; b = 0~9) S62.9aXB (a = 0;1,2)
Hip fracture	820.8; 820.9; 905.3 820.aa (aa = 00;01;02;03;09;10;11;12;13;19) 820.bb (bb = 20~22; 30~32)	S72.0aaA (aa = 01;02;09; 11;12;19; 51;52;59; 91;92;99) S72.0abA (ab = 21~26; 31~36; 41~46; 61~66) S72.0aaB (aa = 01;02;09; 11;12;19; 51;52;59; 91;92;99) S72.0abB (ab = 21~26; 31~36; 41~46; 61~66) S72.0aaC (aa = 01;02;09; 11;12;19; 51;52;59; 91;92;99) S72.0abC (ab = 21~26; 31~36; 41~46; 61~66) S72.1aaA (aa = 01;02;09) S72.1abA (ab = 11~16; 21~26; 31~36; 41~46) S72.1aaB (aa = 01;02;09) S72.1abB (ab = 11~16; 21~26; 31~36; 41~46) S72.1aaC (aa = 01;02;09) S72.1abC (ab = 11~16; 21~26; 31~36; 41~46) S72.2aXA (a = 1~6); S72.2aXB (a = 1~6) S72.2aXC (a = 1~6)
**Comorbidities**		
Myocardial infarct	410.X, 412.X	I21.X, I22.X, I25.2
Congestive heart failure	428.X	I09.9,I11.0, I13.0, I13.2, I25.5, I42.0, I42.5–I42.9, I43.x, I50.x, P29.0
Peripheral vascular disease	443.9, 441.X, 785.4, V434, 384.8	I70.x, I71.x, I73.1, I73.8, I73.9, I77.1, I79.0, I79.2, K55.1, K55.8, K55.9, Z95.8, Z95.9
Cerebrovascular disease	430.X–438.X	G45.x, G46.x, H34.0, I60.x–I69.x
Dementia	290.X	F00.x–F03.x, F05.1, G30.x, G31.1
Chronic lung disease	490.X–496.X, 500.X–505.X, 506.4	I27.8, I27.9, J40.x–J47.x, J60.x–J67.x, J68.4, J70.1, J70.3
Connective tissue disease	710.0, 710.1, 710.4, 714.0–714.2, 714.81, 725.X	I05.x, M06.x, M31.5, M32.x–M34.x, M35.1, M35.3, M36.0
Ulcer	531.X–534.X	K25.x–K28.x
Chronic liver disease	571.2, 571.4, 571.5, 571.6	B18.x, K70.0–K70.3, K70.9, K71.3–K71.5, K71.7, K73.x, K74.x, K76.0, K76.2–K76.4, K76.8, K76.9, Z94.4
Diabetes	250.0–250.3, 250.7	E10.0, E10.l, E10.6, E10.8, E10.9, E11.0, E11.1, E11.6, E11.8, E11.9, E12.0, E12.1, E12.6, E12.8, E12.9, E13.0, E13.1, E13.6, E13.8, E13.9, E14.0, E14.1, E14.6, E14.8, E14.9
Diabetes with end organ damage	250.4–250.6	E10.2–E10.5, E10.7, E11.2–E11.5, E11.7, E12.2–E12.5, E12.7, E13.2–E13.5, E13.7, E14.2–E14.5, E14.7
Hemiplegia	344.1, 342.X	G04.1, G11.4, G80.1, G80.2, G81.x, G82.x, G83.0–G83.4, G83.9
Moderate or severe kidney disease	582.X, 583.0–583.7, 585.X–586.X, 588.X	I12.0, I13.1, N03.2–N03.7, N05.2–N05.7, N18.x, N19.x, N25.0, Z49.0–Z49.2, Z94.0, Z99.2
Tumor, leukemia, lymphoma	140.X–195.X, 200.X–208.X	C00.x–C26.x, C30.x–C34.x, C37.x–C41.x, C43.x, C45.x–C58.x, C60.x–C76.x, C81.x–C85.x, C88.x, C90.x–C97.x
Moderate or severe liver disease	572.2–572.8, 456.00–456.21	I85.0, I85.9, I86.4, I98.2, K70.4, K71.1, K72.1, K72.9, K76.5, K76.6, K76.7
Malignant tumor, metastasis	196.0–199.1	C77.x–C80.x
AIDS	042.0–044.9	B20.x–B22.x, B24.x

**Table A2:** Autoimmune disease.

Autoimmune Disease	ICD-9-CM	ICD-10
Psoriatic arthritis	696.0	L40.5
Rheumatoid arthritis	714.X	M05
Ankylosing spondylitis	720.X	M45
Systemic lupus erythematosus	710.0	M32

**Table A3:** Medication.

Medication	Patients	Medication Days (Means) *
Conventional DMARDs		
Cyclosporin	3283	191
Leflunomide	3079	173
Mycophenolate mofetil	350	224
Mycophenolic acid	210	212
Tocilizumab	2	73
Methotrexate	4782	232
Anti-TNF therapeutics		
Adalimumab	681	157
Certolizumab pegol	0	
Etanercept	723	158
Golimumab	234	158
Infliximab	0	
mTOR inhibitors		
Everolimus	15	283
Sirolimus	27	207
Tacrolimus	181	201
Temsirolimus	0	
Other biologics		
Abatacept	61	177
Ixekizumab	0	
Secukinumab	7	89
Ustekinumab	87	75
Vedolizumab	0	
Natalizumab	0	
Tocilizumab	80	172

*: per year.
